# Restoring vertebrate predator populations can provide landscape‐scale biological control of established invasive vertebrates: Insights from pine marten recovery in Europe

**DOI:** 10.1111/gcb.16236

**Published:** 2022-06-15

**Authors:** Joshua P. Twining, Colin Lawton, Andy White, Emma Sheehy, Keziah Hobson, W. Ian Montgomery, Xavier Lambin

**Affiliations:** ^1^ Department of Natural Resources Cornell University Ithaca New York USA; ^2^ School of Biological Sciences Queen's University Belfast UK; ^3^ School of Natural Sciences, Ryan Institute National University of Ireland Galway Galway Ireland; ^4^ Maxwell Institute for Mathematical Sciences, Department of Mathematics Heriot‐Watt University Edinburgh UK; ^5^ School of Biological Sciences University of Aberdeen Aberdeen UK

**Keywords:** behavioural response, biological control, carnivore recovery, generalist predators, invasive naivety, invasive species, native predators, prey switching, refuge

## Abstract

Invasive species pose one of the greatest global threats to biodiversity. There has been a long history of importing coevolved natural enemies to act as biological control agents to try to suppress densities of invasive species, with historically limited success and frequent adverse impacts on native biodiversity. Our understanding of the processes and drivers of successful biological control has been focussed on invertebrates and is evidently limited and potentially ill‐suited with respect to biological control of vertebrate populations. The restoration of native vertebrate predator populations provides a promising nature‐based solution for slowing, halting, or even reversing the spread of some invasive vertebrates over spatial scales relevant to the management of wildlife populations. Here, we first review the growing literature and data from the pine marten‐red and grey squirrel system in Europe. We synthesise a multi‐decadal dataset to show that the recovery of a native predator has resulted in rapid, landscape‐scale declines of an established invasive species. We then use the model system, predator–prey interaction theory, and examples from the literature to develop ecological theory relating to natural biological control in vertebrates and evolutionary processes in native‐invasive predator–prey interactions. We find support for the hypotheses that evolutionary naivety of invasive species to native predators and lack of local refuges results in higher predation of naive compared to coevolved prey. We apply lessons learnt from the marten‐squirrel model system to examine the plausibility of specific native predator solutions to some of the Earth's most devastating invasive vertebrates. Given the evidence, we conclude that depletion of vertebrate predator populations has increased ecosystem vulnerability to invasions and thus facilitated the spread of invasive species. Therefore, restoration of vertebrate predator populations is an underappreciated, fundamental, nature‐based solution to the crisis of invasive species and should be a priority for vertebrate invasive species management globally.

## Graphical abstract

The restoration of native vertebrate predator populations provides a promising nature‐based solution for slowing, halting, or even reversing the spread of some invasive vertebrates. We find support for the hypotheses that evolutionary naivety of invasive species to native predators and lack of local refuges results in higher predation of naive compared to coevolved prey. Given the evidence, we conclude that depletion of vertebrate predator populations has increased ecosystem vulnerability to invasions and facilitated the spread of invasive species. Restoration of vertebrate predator populations should be a priority for vertebrate invasive species management globally.

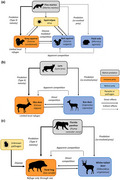

## 1 INTRODUCTION

Over the past 500 years, increasing global occurrence of invasive species has been associated with more vertebrate extinctions than any other factor (Bellard et al., 2016; Seebens et al., 2017). Biological control is a key tool for ameliorating the impacts of invasive species at a landscape‐scale where human‐led control approaches are implausible due to logistical and financial constraints (Van Driesche et al., 2008). The premise for introducing natural enemies from the invasive species' native range is that they will have coevolved with the target species and therefore, should be effective at searching for, finding, and consuming the species of interest (Murdoch et al., 1985). This approach capitalises on the knowledge that natural enemies may limit and regulate the size of prey/host populations (e.g. Krebs et al., 2017) and that introduced species in the absence of natural enemies may reach extraordinary densities, the so‐called enemy release effect (Schulz et al., 2019). However, failures are commonplace highlighting our continued inability to accurately predict: (1) when biological control agents will be effective at limiting and then regulating target species; and (2) when biological control agents will have adverse impacts on native species (Smith et al., 2018). The key challenge in developing a more comprehensive understanding of the mechanisms that underpin successful biological control is discovering general patterns that identify when a natural enemy will regulate an invasive species, without posing a significant risk to non‐target species.

Analyses of the historical biological control record demonstrate a 75% greater chance of successful suppression of an invasive pest using a natural enemy without a shared coevolutionary history with the pest, as opposed to a coevolved species (Hokkanen & Pimentel, 1984, 1989). While there are disputes about the degree of population control achieved when comparing coevolved and novel interactions due to the latent nature of successful invasions (Waage, 1990), this idea highlights the potential importance of evolutionary processes in biological control (Wanger et al., 2010). Historically, there have been several ill‐fated attempts to introduce mammalian predators as biological control agents, including as recently as 1979 when Javan mongoose (*Herpestes javanicus*) were released on Amami Island, Japan (Yamada, 2002). This follows multiple historical introductions of mongoose throughout Hawaii (Hays & Conant, 2007) and various non‐native mustelids from Europe to New Zealand in the hope of controlling introduced co‐evolved European rabbits (*Oryctolagus cuniculus*) and leading to disastrous impacts on native biodiversity that was naïve to the predators (King, 2019). Considering these cases and taking into consideration the potential role of evolutionary processes, it begs the question whether there is a fundamental difference between predator–prey interactions involving novel predators and naive prey species and interactions between coevolved native species that could result in predictably different outcomes.

While most research on biological control has focussed on insects or more narrowly still, parasitoids, many problematic invasive species are mammals. We posit that theory devised on invertebrate models is of limited applicability to vertebrate systems due to invertebrates' lack of centralised brains and reliance on innate, less flexible, behaviours with a limited capacity for learned adaptation (excluding cephalopods, Wells, 1965). Arguably, comparatively higher brain function in vertebrates enables a greater capacity for rapid adaptation to novelty both in predators and in their prey. However, even amongst vertebrates, capacity to adapt appears to vary significantly between taxa. For example, following the invasion of toxic cane toads (*Rhinella marina*) to Australia, sustained predation, and an inability to adapt to the new poisonous prey resulted in declines of up to 77% in freshwater crocodiles (*Crocodylus johnstoni*, Letnic et al., 2008), with declines still being observed over half a decade later (Britton et al., 2013). Mammals, on the other hand, for example, the rakali (*Hydromys chrysogaster*), a native predatory rodent, rapidly adapted novel behaviours in response to this toxic prey. Rakali preferentially predated larger cane toads and only consumed specific non‐toxic organs (Parrot et al., 2019). Temperature‐dependant metabolism explains how the endothermic physiology of mammals (and birds) facilitated the development of brain sizes 4–40 times larger than those of similar‐sized ectotherms (Yu et al., 2014). This increased neural capacity results in significantly higher calorific requirements to sustain the energetically costly tissue (Isler & Schaik, 2006). The higher consumptive requirements of these taxa alongside an increased capacity to adapt to novel situations sets mammals and birds apart from other vertebrates. We posit that this applies both to the challenges presented when attempting to control them when they are invasive species, and in their potential to act as effective natural biological control agents.

Mammalian carnivores are recovering from past persecution in certain locations following legal protection (Chapron et al., 2014). The resulting partially restored carnivore guilds presents unique opportunities for understanding predator prey interactions and potentially managing biological invasions. The lack of formal consideration of vertebrate predators in the biological control literature may reflect the fact that the top predator guild has been strongly affected by human persecution. Indeed, the historically depauperated state of many predator guilds likely contributed to the invasibility of communities by non‐native species lower in the trophic chain (Wallach et al., 2010) such as muskrat (*Ondatra zibethicus*), coypu (*Myocastor coypus*), grey squirrels (*Sciurus carolinensis*), chipmunk (*Eutamias sibiricus*), sika deer (*Cervus nippon*), and raccoon dogs (*Nyctereutes procyonoides*). A growing literature demonstrates the diverse and underappreciated role of predators in shaping ecosystems and has called for renewed restoration efforts to ameliorate or buffer against environmental challenges (Estes et al., 2011; Ritchie & Johnson, 2009, 2012). However, the scope for recovering vertebrate predators to reverse established invasions through predation remains unknown. The generality of the invasive naivety effect, an opposite of the enemy release hypothesis (Wanger et al., 2010), for underpinning native predator population's role in providing resistance and reversal to invasion is yet to be appropriately considered. The invasive naivety effect suggests that evolutionarily naive invasive prey species are unlikely to display appropriate anti‐predator behaviours in response to native predators, resulting in elevated predation and potential for biological control. Unfortunately, extant biological control theory, developed largely on insects and parasitoids, offers little guidance when it comes to vertebrate predators.

It is therefore opportune to extract potentially broadly applicable insights from situations where a recovering native predator exerts effective control on an invasive vertebrate that became established when the predator was absent due to historical persecution. In this review, we use the emerging research on the pine marten (*Martes martes*)–red squirrel (*Sciurus vulgaris*)–grey squirrel system, to illustrate the role that predation by a native predator plays in limiting invasive prey in competing host populations. We use this model system and other examples in the literature to extend natural biological control theory, and to identify key features that may enable carnivores to control invasive species on landscape‐scales, if their currently depleted populations were restored.

### 1.1 Marten–squirrel model system

The pine marten is a small (1–2 kg), semi‐arboreal carnivore, native to Eurasia. An omnivorous generalist throughout much of its range, in Ireland and Britain, the pine marten is a shared predator of two prey species linked by exploitative and disease mediated competition, the native red squirrel and the invasive grey squirrel (Tompkins et al., 2003). Once widespread and abundant throughout Britain and Ireland, the pine marten underwent severe historical decline during the 20th century (Langley & Yalden, 1977; O'Sullivan, 1983), by which time the species range had retracted to the western, seaboard counties in Ireland, and the northerly reaches of the Scottish Highlands in Britain.

The eastern grey squirrel was introduced into Europe in the late 1800s and early 1900s (Bertolino, 2008). Following multiple, deliberate introductions at the turn of the 20th century, when pine martens were largely absent throughout Britain and Ireland, the grey squirrel population expanded at a rate of 0.1–13 km per year (Reynolds, 1985; Teangana et al., 2001), replacing native red squirrels throughout much of their former range (Gurnell et al., 2004; Shuttleworth et al., 2016). Grey squirrels have also been introduced to mainland Europe in Italy and British Columbia in Canada (Bertolino, 2008; McInnes et al., 2020). However, to date, there is no research on the impacts of predation on these invasive populations, or their interactions with native carnivores. The emergent body of research on the interactions of the pine marten, the red squirrel, and the grey squirrel makes them an ideal model system to examine and extend predator–prey ecological theory on the ability of native carnivores to provide natural biological control of invasive species.

### 1.2 Effects of a recovering native predator on naive invasive and coevolved native prey: From local to landscape‐scale impacts

Where biological control involves highly mobile populations, whether it achieves exclusion or persistence of the target prey should be considered not only at a local scale but at the scale of the metapopulation. Scaling up of local impacts to the metapopulation could be countered by asynchronous dynamics of locally independent populations, dispersal rates of both natural enemies and target species, and connectivity within the landscape (Holyoak & Lawler, 1996). The resultant “game of hide and seek” between predators and prey across large, heterogeneous areas is hypothesised to confer a degree of resilience to the prey species, resulting in a time lag prior to exclusion. This was elegantly demonstrated long ago by Huffaker's classic experiment using six spotted mites (*Eotetranychus sexmaculatus*) and predatory mites (*Typhlodromus occidentalis*) (Huffaker, 1958). A similar scenario plays out in the interaction between native water voles (*Arvicola amphibius*), invasive American mink (*Neovison vison*), and non‐native rabbits in Scotland. Here, water voles only persist in metapopulation networks that are distant from established populations of the mink's staple prey, rabbits. Water voles are extirpated by the mink when rabbits occur locally, but areas without rabbit are less profitable and therefore rarely impacted by the predatory mink (Oliver et al., 2009). Thus, a relevant lesson for ecological understanding is that the uneven distribution of strong local impacts across heterogeneous landscapes may preclude or delay eradication of an invasive prey species by a native predator, with transient refuges from predation allowing persistence and reinvasion by a mobile invasive prey.

**FIGURE 1:**
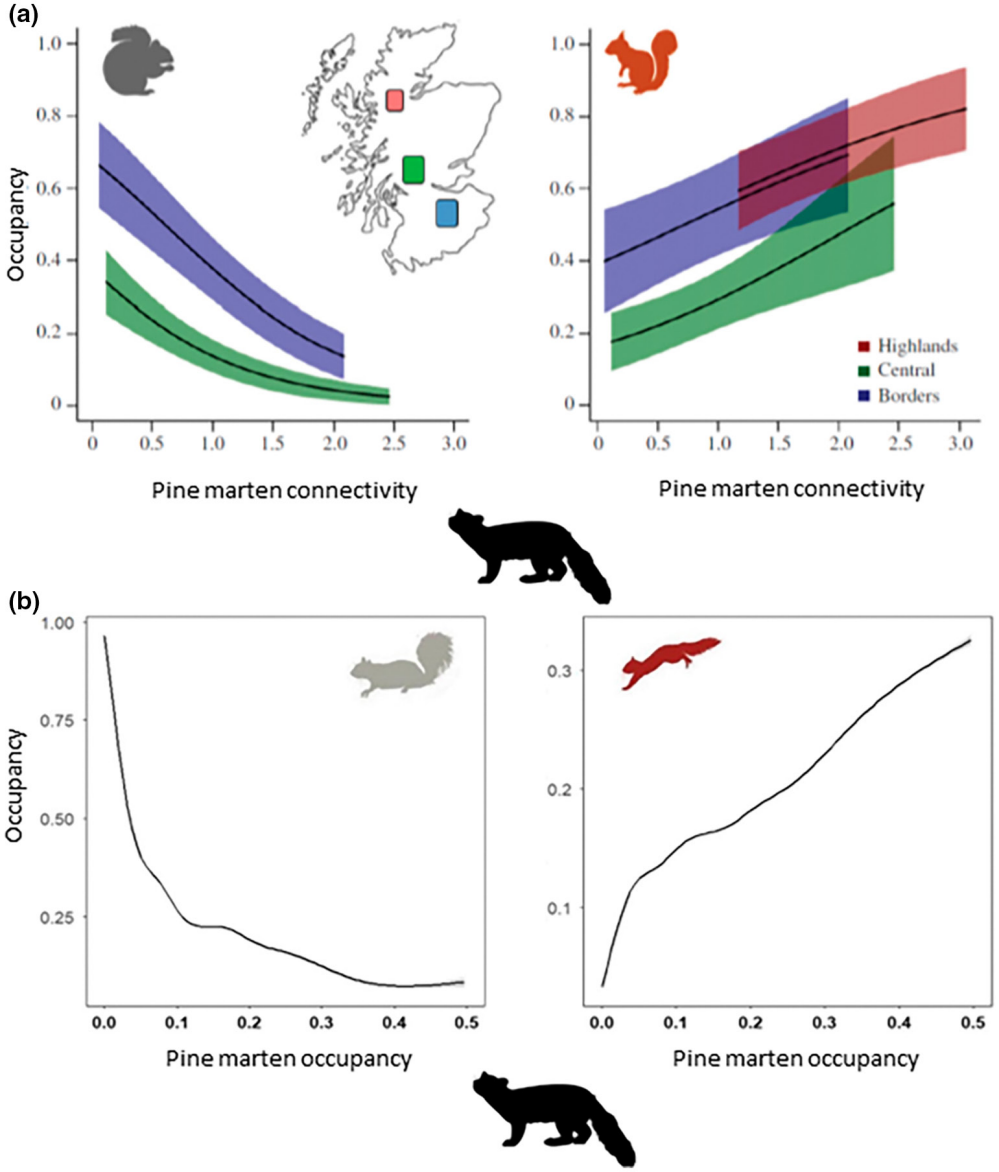
(a) Local scale impacts of pine marten recovery on invasive grey squirrels (left) and native red squirrels (right) in Scotland (Sheehy et al., 2018). (b) landscape‐scale impacts of pine marten recovery on invasive grey squirrels (left) and native red squirrels (right) in Northern Ireland (Twining et al., 2021). Lines are model predictions of the relationship between pine marten and squirrel occupancy.

Research with pine martens and squirrels suggest such a scenario may be occurring (Twining et al., 2021). After legal protection of the pine marten in Britain and Ireland in the 1970s and 1980s, there was a reversal of the population decline (O'Sullivan, 1983; Sheehy et al., 2018; Twining et al., 2021). Building on initial anecdotal reports, a negative spatial correlation between pine marten and grey squirrel occurrence was described prompting the hypothesis that the native predator's recovery was resulting in declines of the long‐established invasive grey squirrel (Carey et al., 2007; Lawton et al., 2015; Sheehy & Lawton, 2014). A quantitative investigation tested this hypothesis in Scotland and showed the impacts of pine marten recovery on invasive grey squirrels to be present across ecosystems (Sheehy et al., 2018). The research demonstrated a negative relationship between local grey squirrel occupancy and marten density weighted connectivity, a metric that describes the intensity of use of a location by marten that considers density in the surrounding area, with even low‐density marten populations depressing local grey squirrel occupancy (Figure 1a, Sheehy et al., 2018). It predicted near extirpation of grey squirrels at a local scale where marten reached the highest density, with concurrent asymmetrical increase in local red squirrel occupancy (Sheehy et al., 2018). While the research predicted landscape‐scale impacts, these were not demonstrated until further quantitative work in Ireland established region‐wide impacts on both grey and red squirrels following pine marten recovery in Northern Ireland (Figure 1b, Twining et al., 2021). Red squirrel recovery across the region was also concomitant with pine marten recolonisation and grey squirrel decline (Twining et al., 2021). However, the single year‐long sampling did not allow characterisation of the spatio‐temporal dynamics resultant from scaling immediate local impacts across a heterogeneous landscape.

**FIGURE 2:**
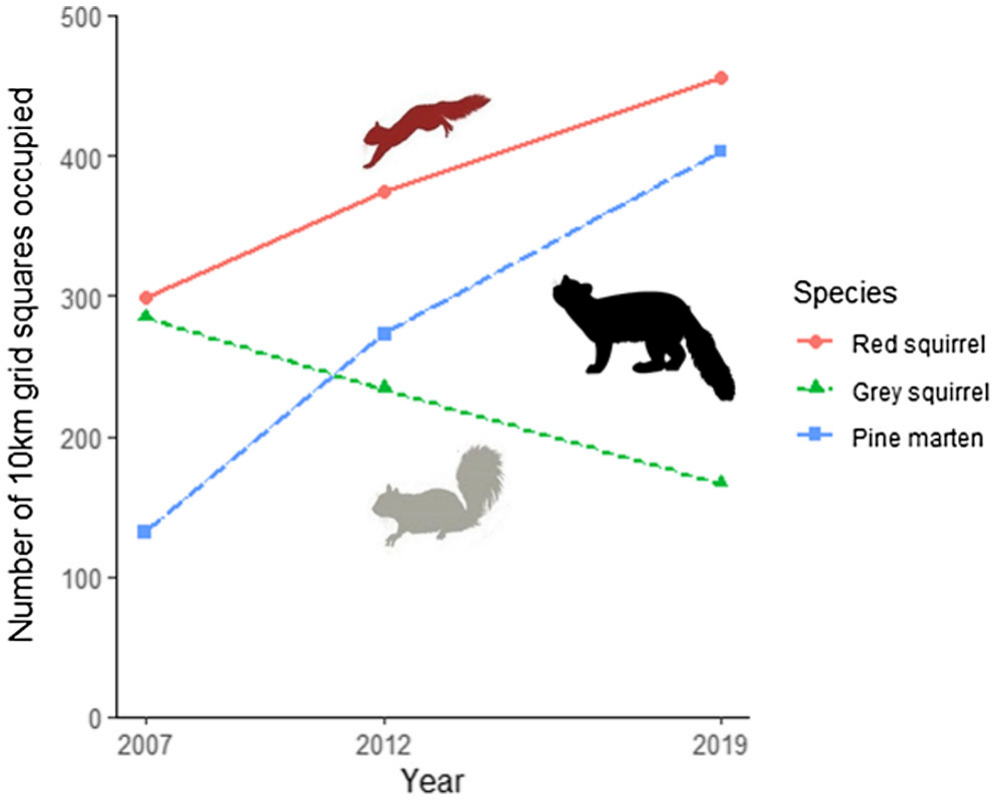
Changes in number of 100 km^2^ grid occupied by the pine marten, the red squirrel, and the grey squirrel over 12 years of monitoring in Ireland (Carey et al., 2007; Lawton et al., 2015, 2020). The blue dashed line is the pine marten, solid red is the red squirrel and dashed green is the grey squirrel.

**FIGURE 3:**
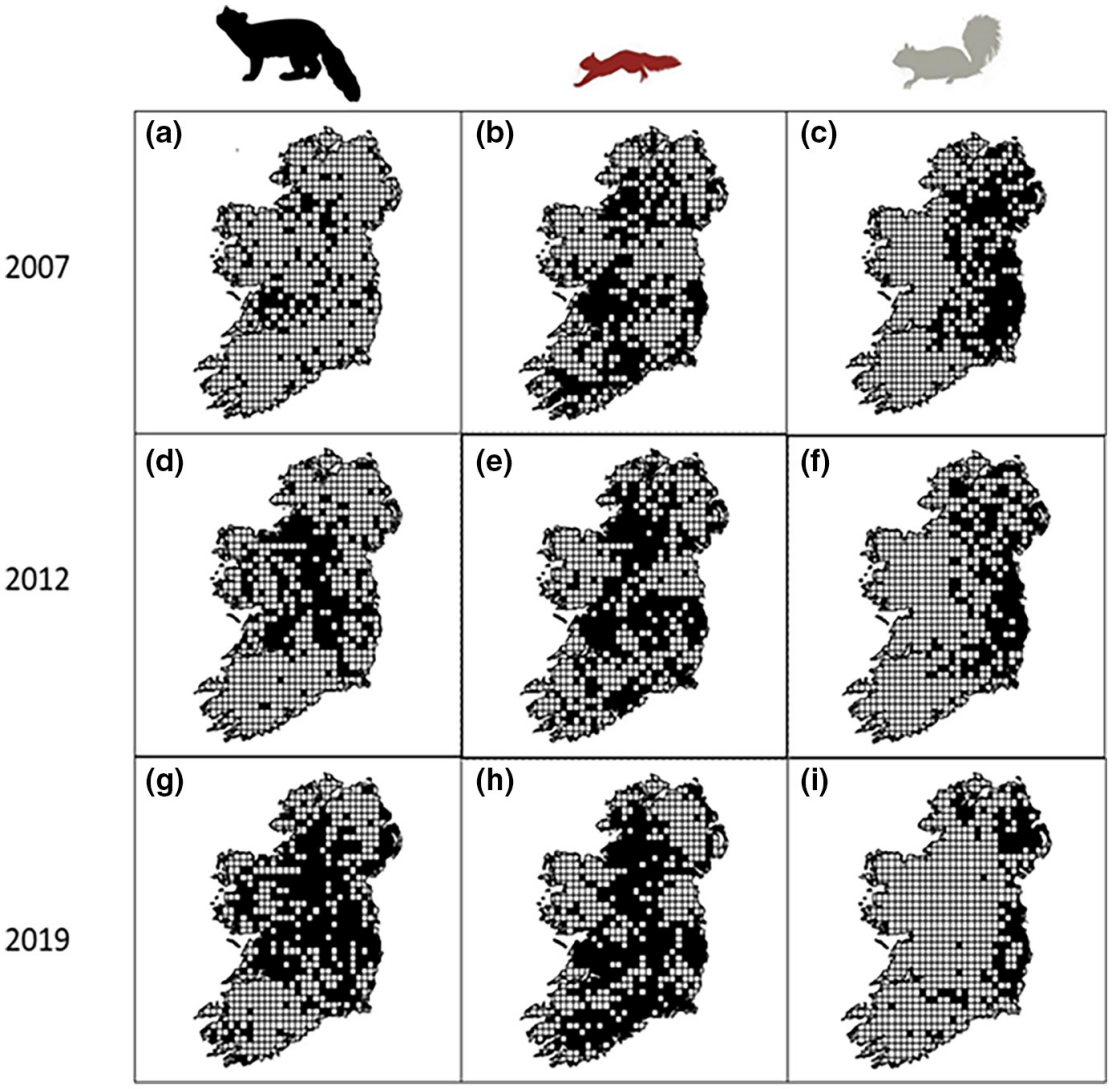
Dynamics of predator recovery on native and invasive squirrels over 12 years in Ireland. Black squares show occurrence of (a) pine marten, (b) red squirrel and (c) grey squirrel in 2007 (Carey et al., 2007); (d) pine marten, (e) red squirrel and (f) grey squirrel in 2012 (Lawton et al., 2015); (g) pine marten, (h) red squirrel and (i) grey squirrel in 2019 (Lawton et al., 2020).

To test for the presence of a lag in the distributional changes of an invasive‐ and a native‐ prey species in response to a recovering shared predator, we collated evidence from a series of distribution surveys conducted from 2007–2019 in Ireland (Figures 2 and 3; Carey et al., 2007; Flaherty & Lawton, 2019; Lawton et al., 2015, 2020). We use these maps with a coarse spatial scale (100km^2^ grid) to examine broad scale patterns of change over a 12‐year period. We conducted our analyses in ArcGIS 10.5 (Environmental Systems Research Institute, Redlands, California) and calculated percentage changes for each of the three species distributions between each survey as:

$$-100\left(1-\frac{\text{current range}}{\text{historic range}}\right).$$

Our analysis of distributional change of the three species indicates that the range of the pine marten has increased in Ireland by 205% from 2007 to 2019, while concurrently red squirrel range increased 52% and grey squirrel range underwent a complementary decrease of 41% (Figure 2). Pine marten and red squirrel range expansions originated from the west of Ireland. They were most pronounced in the midlands and the south of Ireland where grey squirrel declines were greatest (Figure 3). The largest pine marten expansions occurred between 2007 and 2012, with concurrent declines of grey squirrel distribution, with this pattern continuing into the latest period, 2012–2019. Grey squirrels were extirpated from 85% of 100 km^2^ grids where they had co‐occurred with pine marten in 2007 over the 12‐year period (Figure 3).

The absence of an evident lag in range contraction of the invasive prey during predator recovery on a 10 km × 10 km square scale demonstrates the strength of the interaction between a recovering native predator and an evolutionary naive invasive species at that scale (see Figure 2). Immediate local impacts appear to scale up to landscape‐scale, at a 5‐year interval, without the latency in prey range contraction expected if spatial heterogeneity provided resilience to predation. There were relatively synchronised, complementary, changes in distribution of both the invasive and native prey species concurrent with increases in native predator distribution evident in the available time series (Figure 2). The recovery of this native predator leading to the rapid, landscape‐scale decline of a long‐established invasive species suggests recovering carnivore populations have the potential in certain circumstances to control invasive species over large spatial scales. Elucidating the mechanisms by which a native predator effects a decline in an invasive prey species thus benefiting a native prey species, would help identify other carnivore species that may be able to provide successful biological control of invasive species over landscape‐scale following recovery or reintroduction.

### 1.3 Prey switching in predators and vulnerability of coevolved and naive prey

The pine marten is a generalist predator, with a variable diet across its entire range (Twining et al., 2019) and a pronounced prey switching functional response to small mammal density (Zalewski, 2007). Despite variation in prey ingested, the pattern of resource use is consistent throughout the species' entire distribution: seasonal exploitation of ephemerally abundant resources supplemented by a low number of staple prey items (Clevenger, 1993; Helldin, 2000; Pulliainen & Ollinmäki, 1996; Twining et al., 2019; Zalewski, 2007). For example, in Ireland, the pine marten targets birds and squirrels in spring and summer, fruits and invertebrates in autumn and carrion in winter, with wood mice remaining a staple prey item throughout the year (Twining et al., 2019). This capacity for prey switching is observed throughout the species' native range, even where the species acts as a facultative rodent specialist with a diet dominated by voles when they are transiently hyperabundant (*Microtus agrestis* in Scotland, Caryl et al., 2012; *Myodes glareolus* in mainland Europe, Helldin, 2000; Zalewski, 2007). This pattern of resource use conforms to the general understanding of prey switching in predators; where polyphagous species prey switch both seasonally and spatially, targeting resources that are profitable, reflecting their abundance and ease of capture. The density dependence in predator‐induced mortality rate associated with prey switching is the basis for the capacity of predators to regulate prey abundance under some circumstances (Murdoch et al., 1985).

The mechanism underpinning the contrasting effects of the pine marten on two competitively linked prey is hypothesised to be a higher predation rate of the invasive grey squirrels over the native red squirrels (Sheehy et al., 2013; Twining, Montgomery, & Tosh, 2020). However, to limit or exclude a prey population, predation rates of the prey must be sustained over a range of densities, including low prey density (Solomon, 1949). Sustained per capita predation is critical; a lack of prey switching due to strong prey preferences for one prey species, or frequent opportunistic encounters, can limit or even exclude that prey species (Type II functional response, Holling, 1959; Murdoch, 1969). In contrast, predation that focuses on the most abundant prey due to weak preferences can provide refuge for prey at low densities (Type III functional response) and thereby facilitate prey persistence (Holling, 1959; Murdoch, 1969). From our analysis of distributional dynamics above, a type II functional response is expected.

Functional responses have been advocated for as a universal trait of species that can unify invasion ecology (Dick et al., 2017 but see Vonesh et al., 2017). In invasion scenarios, novel invasive predators have been characterized by higher functional responses to naive native prey compared to native predators, that have co‐evolved with their prey. This is driven by significantly lower handling times and thus higher maximum feeding rates (Alexander et al., 2014). The difference in interaction strength between coevolved and novel species pairings has been explained in numerous contexts by the lack of coevolutionary history between invader and native prey (Blackburn et al., 2019; Cunningham et al., 2020; Dick et al., 2014; Ricciardi et al., 2013). If evolutionary processes result in naive prey being more vulnerable to predation than coevolved prey, sustained higher predation rates of more profitable invasive prey, over a range of densities, could be expected. Thus, where a native predator sustains a high predation rate on an invasive prey even at low density, whether by virtue of naivety, ease of capture or short handling time, limitation of the invasive prey populations may ensue.

Recent research has provided evidence of higher consumption of grey squirrels by pine martens comparative to congeneric red squirrels. The invasive competitor is ingested at a rate threefold higher than its native counterpart, with an average 13.8% frequency occurrence (FO) of grey squirrels in the diet of the pine marten (averaged across studies, *n* = 187 scats) compared to a mean 4.9% FO of the red squirrel (*n* = 1199 scats, Sheehy et al., 2013; Twining, Montgomery, & Tosh, 2020). When considering the wider geographic range of the pine marten throughout mainland Europe, where the species co‐occurs with the red squirrel in the absence of the grey squirrel, average frequency of occurrence of the red squirrel in the diet is similar (5.64% FO, *n* = 13,184 scats, Twining, Montgomery, & Tosh, 2020). The predation of grey squirrels occurs exclusively in spring and summer when juveniles are present, and females are restricted to dreys (spring = 14.3% FO, summer = 22% FO, autumn = 0%, winter = 0%, *n =* 155 scats, Twining, Montgomery, & Tosh, 2020). This bi‐modal pattern of predation coinciding with the breeding season of grey squirrels is indicative of the high vulnerability of both females and young at this life stage and conducive to a type‐II functional response arising. New empirical data are required to evaluate this prediction and the associated potential for predation‐driven local extinction. Frequency of occurrence alone lacks essential information (e.g., prey densities) for the estimation of the functional response of the pine marten and assessment of critical thresholds for prey switching that drive a predator's impact on a prey population (Sinclair et al., 1998). Although logistically difficult, research repeatedly assessing population densities of various key prey species alongside quantification of diet over multiple years across different habitat types and varying community compositions would provide much needed knowledge into strength of prey preferences and the drivers of the predator's functional responses.

Despite evident knowledge gaps, an approximately two‐to‐threefold higher predation of invasive species over native species has been reported in other instances of a native vertebrate predator recolonising an area that has been invaded by potential prey in its absence. Whilst opportunities to observe scenarios where native vertebrate predators have recovered and co‐existed alongside novel invasive prey are rare, this trend has been reported in a number of other cases across a variety of different systems including native wolves (*Canis lupus*) consuming twice as many non‐native Corsican moufflon (*Ovis gmelini musimon)* compared to native Chamois (*Rupicapra rupicapra,* Poulle et al., 1997); red‐banded snakes (*Dinodon rufozonatum*) predating three times as many American bullfrogs (*Lithobates catesbeianus*) as native frog species (Li et al., 2011); Celebes toads (*Ingerophrynus celebensis*) limiting invasive yellow crazy ants (*Anoplolepis gracilipes*, Wanger et al., 2010); and neotropical river otters (*Lontra longicaudis*) consuming invasive armoured catfish (*Pterygoplichthys sp.*) on average four times more frequently than the most commonly occurring native fish (Juarez‐Sanchez et al., 2019).

#### 1.3.1 Invasive prey naivety revealed by generalist predator recovery

Anti‐predator behaviours benefit prey species as they reduce the risk of predation, for example, by avoiding predators spatially or temporally, or increasing vigilance while accessing high‐risk areas (Preisser et al., 2005; Sih & Kats, 1991). Observations of elevated predation of invasive prey by native predators have been linked to evolutionary naivety and a lack of appropriate anti‐predator behaviours by the invasive prey. Initially coined the “invasive naivety effect”, from a study on toads and invasive ants, Wanger et al. (2010) proposed that alien prey species may not harbour anti‐predatory defences against novel endemic predators, and this phenomenon may be widespread and as such endemic predators could provide resistance to biotic invasions. The generality of this contention remains unverified in vertebrates. Evidence of native prey naivety against invasive predators is more common (Anson & Dickman, 2012; Anton et al., 2020; Carthey & Banks, 2014; Hudgens & Garcelon, 2011), with rapid extinction or decline of native prey species following predator introductions being attributed to prey naivety (Salo et al., 2007). Naivety is a continuum and Banks and Dickman (2007) proposed the multiple levels of prey naivety framework with three levels defined as: the failure of native prey to recognise a novel enemy (level I naivety), predator recognition but the failure to respond appropriately (level II naivety), or an inherent inability to respond, where the behavioural response of a prey species is ineffective in reducing vulnerability to predation (level III naivety). All three could lead to enduringly high‐predation rates across a range of densities and lead to invasive prey limitation or extinction. As defined here, species lacking naivety would be those adopting behavioural responses against a novel predator that are effective in reducing predation.

In the marten‐squirrel system there are clear differences in the behavioural responses of the native and invasive prey to their shared predator, indicative of naivety playing a decisive role (Sheehy et al., 2018; Twining, Montgomery, Price, et al., 2020). Suggestive indirect evidence emerged from quantification of the propensity of squirrels to visit baited sampling stations in the presence/absence of martens. The lack of variation in detectability of grey squirrels at feeders of various levels of pine marten visitation (*effect of marten presence on detectability*, *β* = 0.74 ± 0.26 [SE], *n* = 101, Sheehy et al., 2018), compared to a strong negative effect of marten presence in the area on red squirrel detectability at feeders (*effect of marten presence on detectability*, *β* = −2.64 ± 0.42 [SE], *n* = 105, Sheehy et al., 2018), led the authors to theorise that the two species may show a different behavioural response to their shared predator (Sheehy et al., 2018). This was tested experimentally in Ireland using camera traps and feeders at 20 sites to observe red‐ and grey squirrel behaviours in the field before and after application of pine marten scent to feeders. Red squirrels reduced their visitation rate to sites applied with marten scent by up to 92% for 48 h (*n* = 2142, Twining, Montgomery, Price, et al., 2020), and when accessing feeders, decreased feeding behaviour by 12% whilst increasing time dedicated to vigilance behaviours fourfold from 4.3% to 17.2% (*n* = 2960 mins of footage, Twining, Montgomery, Price, et al., 2020). Grey squirrels did not show any such behavioural responses. On the contrary, visitations increased on average by 36% following application of pine marten scent, a potential response to a novel stimulus, albeit being highly variable across sites with negligible changes in both feeding and vigilance (*n* = 2150 visits, 1218 mins of footage, Twining, Montgomery, Price, et al., 2020). These results suggest that invasive grey squirrels lack appropriate predator recognition to their shared predator (level I naivety), while the native red squirrels display recognition and effective anti‐predator behaviours.

Thus, we find support for the invasive naivety effect (Wanger et al., 2010), as a mechanism underpinning the ability of native vertebrate predators to provide strong population control of evolutionary naive invasive species. The *invasive naivety effect* proposes that the ability of a native predator to control an invasive species to some degree reflects the evolutionary novelty of the predator. Evidence for this is not limited to the Celebes toad‐yellow crazy ant or marten–squirrel system, advocating for its wider relevance. For example, a study in China observed invasive American bullfrogs to lack appropriate anti‐predator behaviours (type I naivety) to native, red‐banded snakes, leading to a threefold higher predation compared to native anurans that displayed advanced anti‐predator behaviours to the native predator (Li et al., 2011). Preferential predation of the invasive over native species was consistent in this system both in a controlled experimental design and within naturally invaded sites (Li et al., 2011).

As a corollary to the way native predators may acquire a taste for novel non‐native prey (Carlsson et al., 2009) and impact their growth, the longevity of naivety, and hence its value in terms of biological control can be difficult to predict due to its potential to be modified by selection and evolution in prey species. Carthey and Banks (2014) theorise that the best predictors of longevity relate to the evolutionary novelty of the predator to its prey, the suite of defences currently used by the prey, and the degree of specialisation of recognition templates. Even if the prey species do adapt behavioural responses to the predation threat, these behavioural changes do not always lead to reduced population impacts, as prey can remain susceptible to predation (level III naivety, Banks & Dickman, 2007). For example, island foxes (*Urocyon littoralis*) shifted their activity patterns shortly after colonisation by golden eagles (*Aquila chrysaetos*) in California but this did not stop the golden eagle‐driven population declines in island foxes (Hudgens & Garcelon, 2011). If grey squirrels learned to recognise the threat posed by pine martens, and adopted appropriate behavioural responses, this would not preclude pine martens from accessing grey squirrels' arboreal breeding dreys and depressing population growth rates. Evidence from an observational study using biologging of grey squirrels co‐existing with recently reintroduced pine martens in Wales, UK suggests changes in short‐term ranging behaviour by male, but not female grey squirrels in response to pine marten presence (McNicol et al., 2020). It is striking that pine marten densities in this study were much lower than the minimum density where declines in grey squirrel populations have been detected (Sheehy et al., 2018). Thus, this may be an instance of type III naivety, where anti‐predator behaviours are present but ineffective, however, this remains speculative. Thus, in the face of accumulating evidence, we propose that Wanger et al. (2010)'s concept of invasive naivety effect is an underappreciated basis for some native predators' ability to provide biological control of invasive species. Past under appreciation likely reflects that it is only evident when a predator recolonises its native range following the establishment of an invasive species.

#### 1.3.2 The role of local refuge availability for invasive prey in driving native predator impacts

The ability of prey to avoid predation through use of structural or spatial refuges not accessible by the predator, is a key determinant of predator–prey dynamics with the magnitude of impact from predation dependent on the proportion of prey that utilises refuges (Berryman & Hawks, 2006; Sih, 1987). The evidence we review suggests that non‐native prey species that lack spatial refuges, are strongly affected by recovering native predators.

The arboreality and slender, elongate form of the pine marten allows it to access the refuges of squirrels, including dreys (arboreal nest made of branches) and tree cavities, a trait not shared by any other predators of squirrels in Ireland and Britain. The seasonal predation aligning with the breeding seasons of both squirrel species is indicative of martens targeting female and juvenile squirrels when they are restricted to their nests in dreys and tree cavities (Twining, Montgomery, & Tosh, 2020). Pine martens search pattern appears to change in spring and summer, switching their focus to arboreal foraging, searching for nests of birds, consuming adults, fledglings, and eggs when they are abundant and vulnerable (Twining et al., 2019). This prey searching behaviour and the high attack rate on grey squirrels are likely consequences of the ease of capture of prey without a refuge. Red squirrels also use dreys but being around half the size of grey squirrels, they may have access to a wider range of spatial refuges in the form of smaller nesting cavities, as well as the terminal ends of branches inaccessible to martens. Indeed, grey squirrels are approximately 179%–238% of the mass of red squirrels (Kenward & Tonkin, 1986) and have a 24% larger zygomatic arch width on average (red squirrel, *n* = 33, Finnegan et al., 2009; grey squirrel, *n* = 281; McGowan et al., 2014). In line with natural history reports, we posit, red squirrels' low weight and agility enable them to use smaller cavities and the terminal ends of branches to act as crucial refuges from marten predation not available to the invasive grey.

Other studies provide further evidence of elevated predation on non‐native species lacking refuges from native predators. For example, following natural recovery of wolves in the Mercantour mountains of France in the 1990s, ungulate remains were found in 97% of wolf scats. The non‐native Corsican moufflon occurred at twice the frequency of the next most common item, the native chamois, throughout spring, autumn, and winter (Poulle et al., 1997). The higher predation of the non‐native moufflon was hypothesised to be due to the moufflon's inexperience and inability to traverse the difficult topography of the mountains, especially during deep snow cover. Thus, moufflon tended to stay at lower altitudes compared with the native chamois which had access to refuges higher up the mountainside that could not be reached by the wolf (Poulle et al., 1997). Like grey squirrels, Corsican moufflon were first driven into decline, then local extinction by wolf predation (Espuno, 2004). In both cases, the ability to access refuge is limited by morphology and physiology resulting in type III naivete. If refuges from a novel predator are scarce, the development of anti‐predator behaviours will not alter the susceptibility of a prey species to predation due to ease of capture. Thus, we suggest that while invasive naivety may result in elevated predation rates on relatively shorter time scales, the inability of an invasive species to access effective refuge from predation by a native predator is a strong predictor for biological control to occur.

### 1.4 Evaluating the potential impacts of native vertebrate predators and likelihood to provide control of invasive species following restoration

Cases where interactions between natural enemies and their prey are unstable, deterministically leading to the eventual extinction of the prey species are, by definition, rare. It is rarer still that these processes are documented in the same manner as the developing interaction between pine marten and grey squirrel in Britain and Ireland has been documented. The relative contributions of a range of processes remains undetermined even in well studied systems. It is not surprising, therefore, that our understanding of the necessary conditions for the return of a native predator to result in effective biological control of an invasive prey remains fragmentary. Key features of the marten–squirrel system include evolutionary naivety, a lack of access to spatial refuges on behalf of the prey, seasonal prey switching with incidental, opportunistic predation of both adult and juvenile classes, and faster than expected changes in distribution, suggesting a strong interaction. Patterns are consistent with predator–prey theory, suggesting no wholly new concepts are required to explain the outcome of natural biological control observed between marten and grey squirrels or indeed with wolf and Corsican mouflon, or the Celebes toad and the yellow crazy ant. Yet, predicting the outcomes of other new interactions resulting from natural or assisted carnivore recoveries remains challenging. It is pertinent to explore the features of other instances, where conservation interventions could lead to novel circumstances in which hitherto absent predator–prey interactions between native and invasive species might be instated.

**FIGURE 4:**
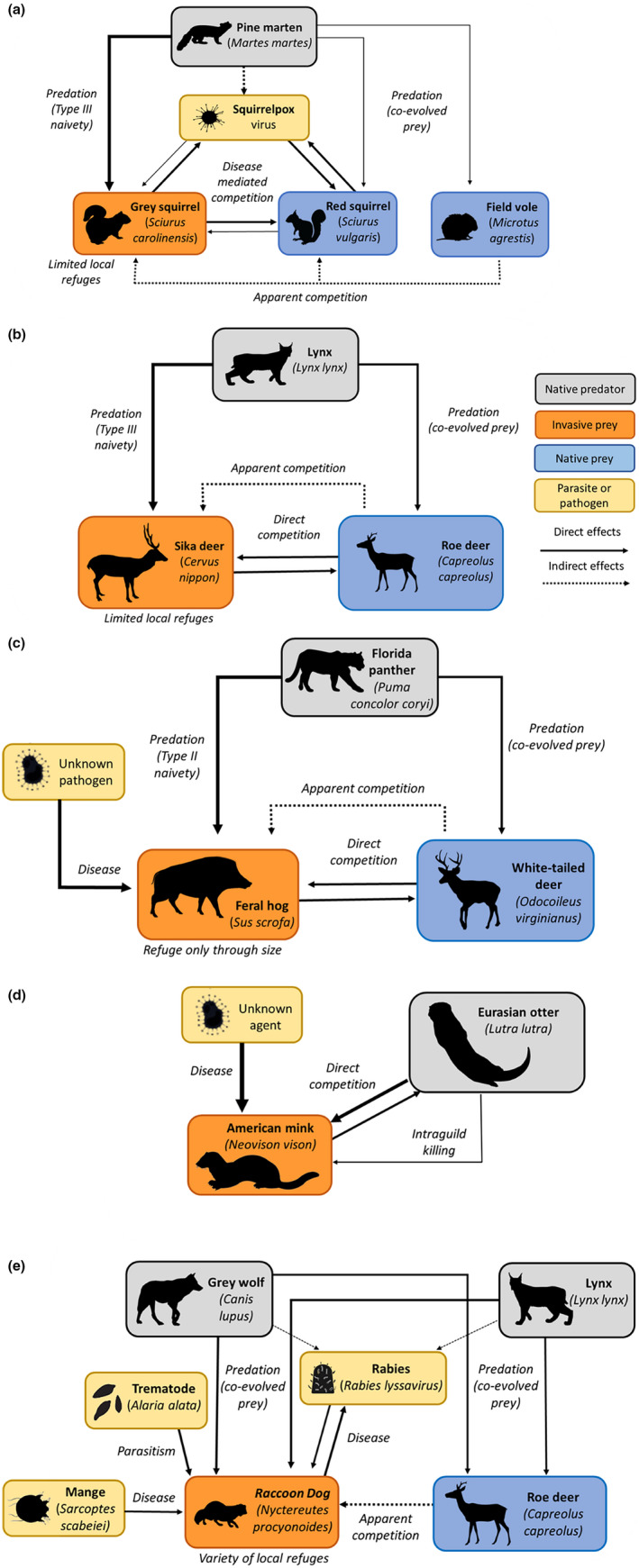
Diagrammatic illustration of the five case studies presented. (a) Marten–squirrel–pox system, (b) lynx–deer system, (c) panther–hog system, (d) otter–mink system, (e) wolf–lynx–raccoon dog–rabies system. Solid lines show direct effects, dashed lines show indirect effects. Grey boxes show native predators, orange boxes invasive species, blue boxes native prey species and gold boxes shows parasites and pathogens. Line width indicates strength of interactions.

Here, we critically examine a variety of case studies both past and present that differ mechanistically from one another and attempt to evaluate the likelihood of success or failure as determined by applying the key features extracted from the marten‐squirrel case study. We now include the impact of pathogens and disease where these have been reported. Hence, the role of squirrel pox in the interaction between red and grey squirrels (Chantrey et al., 2014) has been added to the marten‐squirrel case study (Figure 4a). We use this as an opportunity to highlight a number of factors absent in the marten‐squirrel case study that may underpin why not all predator recoveries will deliver solutions for invasive species, but nevertheless, may limit invasive populations and their impacts without exclusion.

### 1.5 Predator–prey case studies

#### 1.5.1 The Eurasian lynx (*Lynx lynx*) and the sika deer (*Cervus nippon*)

Native to East Asia, sika deer have established and become invasive in many areas globally due to their effects on landscapes through grazing and interactions with native wildlife (Gill & Fuller, 2007; Kalb et al., 2018). The Eurasian lynx is an apex predator, once widespread throughout Europe. Populations of lynx historically declined due to habitat loss, prey declines, and human persecution resulting in the species being extirpated from most of their former range by the turn of the 20th century (Linnell et al., 2009). In temperate forests, small ungulates constitute the bulk of lynx prey (Jędrzejewski et al., 1993), forming on average 91% of biomass consumed annually (Okarma et al., 1997). It is evident that on a continental scale, small deer species densities are lower where lynx are present, and impacts are greater in less productive systems (Melis et al., 2009). Although, often considered specialists, lynx have a broad diet and feed on a wide variety of prey with a demonstrated capacity for prey switching with a focus on ungulate species (Krofel et al., 2011; Odden et al., 2006). Lynx predation is the main source of mortality in roe deer (*Capreolus capreolus*), able to exceed recruitment (Okarma et al., 1997), thus limiting populations.

Little is known about the functional response of lynx to roe deer abundance and their propensity to predate the larger sika deer, itself smaller than red deer which are also preyed upon by lynx. How lynx would apportion their predation between deer species remains unknown and is key to predicting population impacts of lynx on sika deer should lynx populations be restored where sika deer occur. Examining functional responses of lynx in real world experiments in populations in Germany (high density roe deer population, mean 8.4 per km^2^); and potential future populations in Britain (medium density roe deer population, mean 2.22 per km^2^, with sika deer densities ranging from 2.3 to 69.3 per km^2^) and Ireland, (roe deer absent, Burbaitė & Csányi, 2009, sika deer densities ranging from 14 to 44 per km^2^, Swanson & Putman, 2009), would provide insights into the impacts of predator‐mediated apparent competition on functional response and resultant population impacts on persistence of invasive deer species.

The sika deer may utilise habitats with lower predation risk as refuges in response to lynx predation, but the likelihood of this seems minimal when considering the limited effect of predation risk on roe deer habitat selection in a study where 65% of collared deer were confirmed to be predated by lynx (Samelius et al., 2013). As large herbivores, sika deer lack access to fine‐scale refuges from lynx predation that are available to small mammal prey. Additionally, sika deer are expected to display level I naivety to the Eurasian lynx owing to having evolved in the absence of any large felid predators throughout much of its native range (e.g. Japan). The shared features with the marten–squirrel system suggest that lynx have high potential to limit sika deer populations under certain conditions (e.g. low roe deer density).

Despite outstanding knowledge gaps, there is strong evidence to support lynx restoration in systems which are greatly affected by established sika deer populations (if it was deemed socially acceptable) such as Britain, Ireland, Denmark, Germany, and Czech Republic, with impacts expected to be greatest where roe deer densities are relatively low (Britain), or absent (Ireland, see Figure 4b).

#### 1.5.2 The Florida panther (*Puma concolor coryi*) and the feral hog (*Sus scrofa*)

Florida panthers are an apex predator that have recovered from an estimated 20–30 individuals at the end of the 20th century to a population with an upper limit of approximately 230 individuals as of 2015 (FWC, 2017; McBride et al., 2008). Despite the population remaining small in a single isolated population which covers <5% of their historic range (de Kerk et al., 2019), there is emerging anecdotal evidence to suggest that panther recovery may be linked to a localised decline of one of the world's most damaging invasive species, the feral hog. This conjecture appears a direct parallel to the initial anecdotal reports of grey squirrel decline following pine marten recovery in Britain and Ireland (Carey et al., 2007).

Harvest data suggests that feral hog populations have declined locally in the Big Cypress National Preserve since the mid‐1990s (Caudill et al., 2019), which coincides with panther restoration efforts. Whilst feral hogs' vulnerability to fluctuations in water levels in tropical climates and the potential of an unknown disease outbreak provide alternate explanations for observed local declines (Caudill et al., 2019); data on the diet of the panther in Florida also offer a plausible mechanism for predator limitation contributing to declines of this abundant ungulate prey. Large prey, specifically feral hog, dominate the diet of the Florida panther (Caudill et al., 2019; Maehr et al., 1990). Initial work showed that invasive feral hogs occurred in 42% of scats compared to the next common prey item, the native white‐tailed deer in 28% of scats (*n* = 270, Maehr et al., 1990). More recent research showed populations in the northern portion of their breeding range to consume twice the number of feral hogs than white‐tailed deer, whilst also demonstrating distinct spatial differences in diet, with the trend being reversed in the south of their range (*n* = 220, Caudill et al., 2019). This temporally and spatially shifting diet is clear evidence of the prey switching capabilities of the Florida panther. However, anecdotal reports of local declines in both feral hogs and white‐tailed deer, may suggest that the high predation rates of the invasive are not sustained at low prey densities, indicative of a type III functional response, which would not result in exclusion of the invasive prey. Considering the low population size and observed densities of this native predator (1.51 ± .81 per 100 km^2^, Sollmann et al., 2013), and the extreme abundance of feral hogs (Giuliano, 2016), it is likely that predator saturation without prey suppression is a far more plausible outcome in this scenario.

Owing to their size (typically, 70–90 kg in North America, Giuliano, 2016), adult feral hogs are only susceptible to predation by the largest vertebrate predators. However, other than through extremes in their size, feral hogs lack refuges inaccessible to the panther. Adopting a weekly ungulate kill rate of 0.9 (Janis & Clark, 2002), and an averaged FO of wild boar across the northern and southern part of their range of 20.2% (Caudill et al., 2019), the upper estimates for the current population of Florida panthers (230 individuals) is estimated to kill approximately 2174 wild boar a year. Whilst local level impacts are plausible when in conjunction with other drivers (e.g. disease outbreak), the population of wild boar in Florida has been roughly estimated at over 500,000 (Giuliano, 2016). Given the limited range of the panther, predation from the panther population is very unlikely to make a substantial contribution to control of the wild boar at a landscape‐scale in the short term. Whilst the high densities and fecundity of feral hogs may preclude significant population impacts through predation alone, a great deal remains undetermined in this case study. The parallels with several key features of the marten–squirrel system warrant further exploration (see Figure 4c). The potential of predation satiation to limit the propensity of a low density recovering vertebrate predator to control an invasive species as seen is this case study is an important consideration in other systems.

### 1.6 Apex‐mesopredator case studies

#### 1.6.1 Eurasian otters (*Lutra lutra*) and American mink (*Neovison vison*)

The American mink is an invasive semi‐aquatic mustelid, native to North America, which is now established in many countries across South America and Europe. Presence–absence surveys in Britain commencing in the 1970s revealed that during the late 1980s and early 1990s, the number of sites where mink sign was being detected had decreased, concomitant with evidence of recovery of the native Eurasian otter following reintroductions (Bonesi & Macdonald, 2004). A similar decline of mink was observed in Sweden following a sharp population increase in the early 1980s which was attributed to a population crash in red foxes (*Vulpes vulpes*) resultant from a sarcoptic mange outbreak (Carlsson et al., 2010). The hypothesis in Britain was that the larger otter, was outcompeting mink, causing a dietary niche shift, and driving declines of the established invasive. This was supported by correlations between increases in signs of otter occurrence and decreases in mink signs of occurrence in England (Bonesi & Macdonald, 2004; Bonesi et al., 2004, 2006). Similarly in Sweden correlations in the harvest rates of mink and red fox were suggested to be evidence of partial biotic resistance from competition with the dominant red fox (Carlsson et al., 2010). However, in Britain the evidence of mink decline was short lived and was also observed where otters had always been abundant suggesting a separate unidentified cause of the decline in mink abundance. Indeed, later studies in England found mink to co‐exist with otters at all scales through dietary shifts and temporal avoidance (Harrington et al., 2009).

The otter‐mink case study does not meet any of the criteria set out by the marten‐squirrel study (see Figure 4d); otter predation was not a common cause of mortality in mink (despite reported evidence of direct aggression, Bonesi et al., 2000); mink live alongside and co‐exist with the ecologically analogous river otters in their native range (*Lontra canadensis*); and mink as generalists were able to avoid direct competition with otters by switching to terrestrial prey (Bonesi et al., 2004). The case study acts as caution against over‐optimism when examining the potential of recovering native predator to limit an established invasive species in the absence of mechanistic evidence on how a native predator could drive declines of an invasive species. Specifically, it appears circumstances when intra‐guild killing of mesopredators by apex predators are sufficiently strong to result in population limitation of the mesopredator are rare (Waggershauser et al., 2021, but see Hudgens & Garcelon, 2011).

### 1.7 The raccoon dog (*Nyctereutes procyonoides*) and interactions between multiple predators, disease, and apparent competition

Raccoon dogs are medium‐sized carnivores native to East Asia and are one of the most successful non‐native carnivorans in the world. The species quickly colonised new areas after being introduced into the European part of the former USSR in the early 1900s (Helle & Kauhala, 1991). Today the raccoon dog is widespread throughout northern and eastern Europe and continues to spread through Central Europe (Kauhala & Kowalczyk, 2011). They serve as an important host reservoir for rabies with growing concern that the species will reintroduce the disease to rabies‐free areas with serious implications for both animal and human health (Singer et al., 2008).

The raccoon dog has exceptionally high reproductive outputs, with a mean litter size of 8–10 individuals (Kowalczyk et al., 2009). Multiple radio‐tracking studies have shown seasonal intra‐guild predation of both adults and juveniles by lynx and wolves to be the leading cause of mortality in invasive raccoon dog populations (53% of collared animals, *n* = 64, Sidorovich, 2011; 40% of collared animals *n* = 15, Kowalczyk et al., 2009). Raccoon dog's susceptibility to predation has been linked to their reliance on carrion, with the remains of kills made by larger carnivores being a very important component of their diet, especially in winter (Jędrzejewska & Jędrzejewski, 1998). Carrion provisioning by large carnivores can enhance suppression of mesocarnivores through increased incidence and intra‐guild killing (Prugh & Sivy, 2020). The raccoon dog co‐occurs with the Eurasian lynx and the wolf in part of its native range; thus, naivety is not expected. Despite high levels of mortality, predation alone appears to have minimal impacts on healthy raccoon dog populations. This has been attributed to their high fecundity, access and use of burrows as refuge, and hibernation in cold climates which effectively removes them from the system for 5 months of the year (Kauhala & Kowalczyk, 2011; Kowalczyk et al., 2009; Sidorovich, 2011). This suggests that high fecundity linked with availability of spatial refuges may preadapt this species against the typically high population impacts resultant from seasonal predation of both adults and young.

When disease and predation interact together to impact a host/prey population, the outcomes are often difficult to predict; the effects can be subadditive thereby reducing the impact on the target population and resulting in increases or stabilisation of target population (e.g. Hudson et al., 1992), or superadditive, resulting in severe decline. Theoretical and emerging empirical evidence suggest the resultant impact depends on numerous factors including parasite‐induced vulnerability to predation, trait‐mediated effects, and the transmission function of the parasite (Hatcher & Dunn, 2011). Population dynamics of raccoon dogs in Belarus are characterised by a 40‐year cycle, with cyclicity thought to be driven by infestations of a trematode, *Alaria alata*, and sarcoptic mange (Rotenko & Sidorovich, 2017). While mange is a globally distributed disease (Astorga et al., 2018), *A. alata* is reported to be a species of European carnivores (Korpysya‐Dzirba et al., 2021). We posit the generality of the invasive naivety effect applies to pathogens as well as predators, and that raccoon dog's suspected naivety to this trematode may increase their susceptibility to its adverse impacts and potential to drive declines. At the peak in the population cycle (1997–1999), average population density was estimated as 18.9 individuals per 10 km^2^. The density then dropped to 6.8 individuals per 10 km^2^ in an initial nadir in the population cycle (2003–2004). This was followed by a subsequent decline, coinciding with population crashes of roe deer, the primary prey of lynx and wolves in March–April 2013, the raccoon dog population was observed to be nearly exterminated by heavy predation from wolves and lynx, with densities dropping to 0.6 individuals per 10 km^2^ (Rotenko & Sidorovich, 2017). In this example, high density populations of co‐evolved apex predators interacted with disease‐mediated population cycles to supress an invasive species population, establishing a lower stable state during periods of prey scarcity due to apparent competition (see Figure 4e). While local extinction of populations may not be possible, models have shown that rabies is unlikely to spread in populations of raccoon dogs where densities are low (Singer et al., 2008), and thus, management strategies that can achieve suppression of raccoon dog populations are of huge value to conservation efforts and human health.

This case study not only demonstrates the critical importance of maintaining diverse and healthy predator guilds in controlling invasive populations and disease, but it also highlights additional outstanding knowledge gaps. Competition, predation, and disease interact dynamically to drive species persistence and community assemblages. Yet, we lack both the theoretical and empirical understanding of the indirect interactions between these processes, and their importance in determining species co‐existence, exclusion, and community formation. Bridging this gap will enable truly integrated, evidence‐based, management strategies using predation, disease, and competition in combination to provide control of the most problematic and persistent of invasive species.

## 2 CONCLUSIONS

**FIGURE 5:**
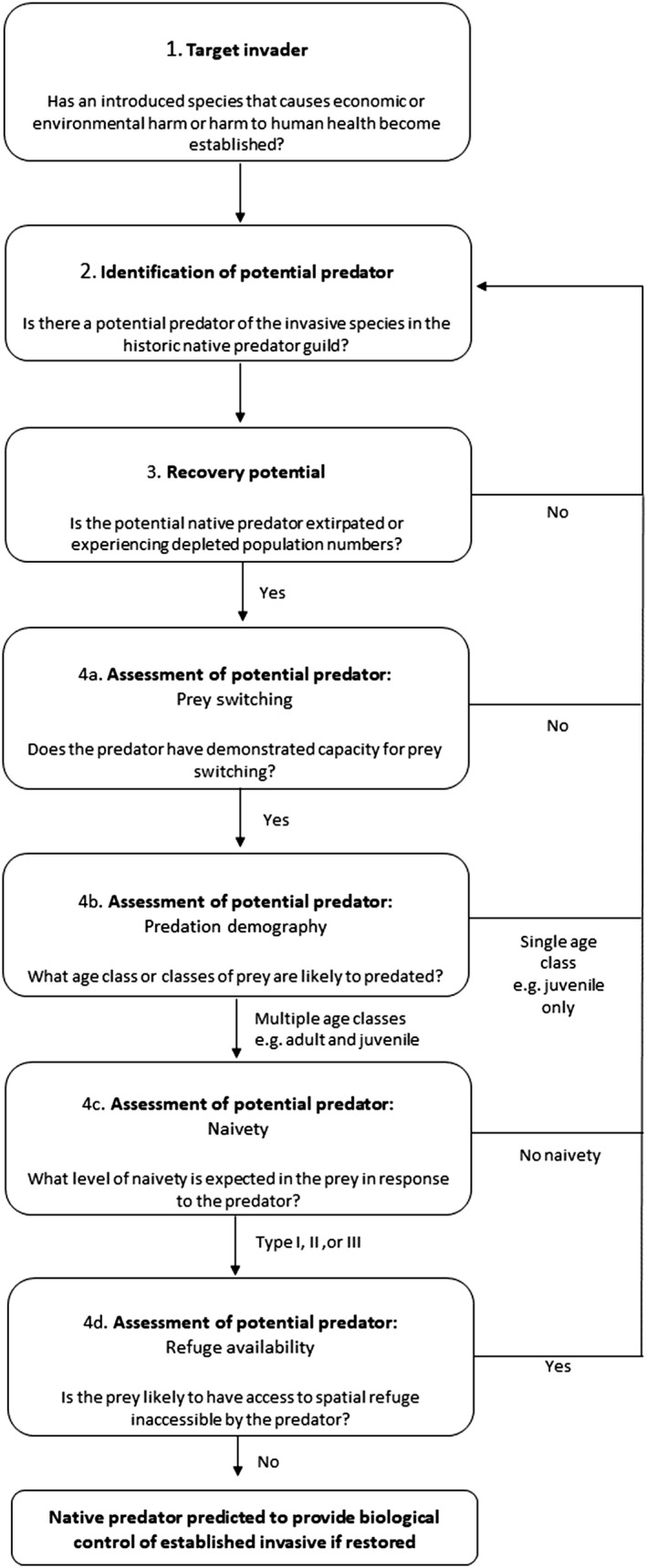
A decision tree providing a general framework for identifying and assessing the potential of a native predator to provide control of an established invasive species should the species be restored or reintroduced.

1. The range of case studies reviewed here demonstrate the potential role native vertebrate predators could play in providing biological control of certain established invasive vertebrates over a landscape‐scale following restoration. Both empirical and theoretical data derived from the marten–squirrel system and others suggest that globally depleted predator guilds may have facilitated the establishment and spread of invasive vertebrates. The observed lack of latency in distributional decline of an established invasive species following native predator recovery highlights the potential for rapid, landscape‐scale, control of some of the most harmful vertebrate invasive species following the reintroduction or restoration of certain native predators. Thus, we propose supporting and restoring native predator populations will prove a valuable management strategy for invasive species globally.
2. The case studies examined provide evidence to support the theory that predation of evolutionary naive prey by a novel predator is likely to result in higher population level impacts compared to that of coevolved prey, especially if the prey species lacks refuge from predation. This is likely due to shorter handling times and thus higher functional responses, with an observed trend towards higher frequency of occurrence of naive invasive prey over native prey in the diet of native predators. However, critical mechanistic data that underpins a predator's capacity to limit invasive prey in different real‐world landscapes remains limited. Thus, there is a pressing need for empirical evidence from applied contexts on the consequences of prey switching and predators' functional responses on prey populations.
3. The ability of a native vertebrate predator to provide rapid, strong, control of an invasive species likely hinges on six key features extracted from the marten–squirrel system and other case studies examined, that we propose can be used to assess likelihood of success prior to predator restoration: (a) the predator's capacity to prey switch to the novel prey; (b) the range of age classes likely to be predated; (c) the level of naivety presented by the invasive species to a novel predator leading to sustained predation; (d) the prey species' access to spatial and temporal refuges inaccessible by the predator; (e) the comparative density between the predator and the prey; and, (f) the trophic position of both the predator and prey, with impacts more likely on true prey species as opposed to between apex and mesopredators. (a)–(d) are drawn from the marten–squirrel system, whilst (e) and (f) emerge from the wider case studies explored. We use these features to produce a conceptual framework for assessing the potential of native predators to provide control of an established invasive species (see Figure 5). The critical question of whether the impacts of invasive naivety on prey are likely to be transient and countered by adaptation hinges on identifying morphological and physiological constraints in adaptation of novel behaviours in naive prey; and using these factors to predict how effective novel behaviour adaptation can be in reducing predation rate.
4. We provide evidence on the processes at work and hence additional ecological justification for the proposed restoration of native predators such as the Eurasian lynx, the Florida panther and the wolf (e.g. Ritchie et al., 2012) as a means to reduce the impacts and spread of some of the most harmful invasive vertebrates globally. We argue that natural biological control may prove a more predictable and less risky alternative to further importation of novel species for biological control. However, we recognise a lack of long‐term quantitative data from applied systems that display pronounced spatial dynamics, habitat heterogeneity, and temporal stochasticity precludes robust predictions of natural biological control outcomes in real‐world landscapes. Empirical validation of theory on the role of temporal and spatial refuges on multi‐trophic system outcomes from initial restoration and reintroduction attempts will increase our ability to predict how these factors can lead to large‐scale coexistence of strongly interactive species, and thus guide future natural biological control efforts. Further evidence from the proposed case studies would bridge some of our largest knowledge gaps in predicting biological control that stem from a failure to consider and understand the dynamic indirect interactions of predation, disease, and apparent competition, which if developed, could form the foundation of integrated approaches to tackle the most difficult and problematic of invasive species.

### AUTHOR CONTRIBUTION

JPT, WIM, CL, and XL conceptualised the review, CL, JPT, ES contributed data to the review, JPT conducted analyses, JPT wrote the first draft. All co‐authors contributed meaningfully to revisions.

## OPEN RESEARCH STATEMENT

Data are already published and publicly available, with those items properly cited in the submission. This submission contains no novel code.

## Data Availability

Data are already published and publicly available, with those items properly cited in the submission. This submission contains no novel code.
